# Cause-specific mortality estimates for Malaysia in 2013: results from a national sample verification study using medical record review and verbal autopsy

**DOI:** 10.1186/s12889-018-6384-7

**Published:** 2019-01-24

**Authors:** Azahadi Omar, Shubash Shander Ganapathy, Mohamad Fuad Mohamad Anuar, Yi Yi Khoo, Chandrika Jeevananthan, S. Maria Awaluddin, Jane Ling Miaw Yn, Chalapati Rao

**Affiliations:** 1 Institute for Public Health, Ministry of Health Malaysia, Jalan Bangsar, 50590 Kuala Lumpur, Malaysia; 2 Department of Social and Preventive Medicine, University of Malaya, Kuala Lumpur, Malaysia; 3 Department of Global Health, Research School of Population Health, Australian National University, Canberra, Australia

**Keywords:** Mortality, Cause of deaths, Malaysia

## Abstract

**Background:**

Mortality indicators are essential for monitoring population health. Although Malaysia has a functional death registration system, the quality of information on causes of death still needs improvement, since approximately 30% of deaths are classified to poorly defined causes. This study was conducted to verify registered causes in a sample of deaths in 2013 and utilise the findings to estimate cause-specific mortality indicators for Malaysia in 2013.

**Methods:**

This is a cross-sectional study involving a nationally representative sample of 14,497 deaths distributed across 19 districts. Registered causes of deaths were verified using standard medical record review protocols for hospital deaths, and locally adapted international standard verbal autopsy procedures for deaths outside hospitals. The findings were used to measure the validity and reliability of the registration data, as well as to establish plausible cause-specific mortality fractions for hospital and non-hospital deaths, which were subsequently used as the basis for estimating national cause-specific mortality indicators.

**Results:**

The overall response rate for the study was 67%. Verified causes of 5041 hospital deaths and 3724 deaths outside hospitals were used to derive national mortality estimates for 2013 by age, sex and cause. The study was able to reclassify most of the ill-defined deaths to a specific cause. The leading causes of deaths for males were Ischaemic Heart Disease (15.4%), Cerebrovascular diseases (13.7%), Chronic Obstructive Pulmonary Disease (8.5%) and Road Traffic Accident (8.0%). Among females, the leading causes were Cerebrovascular diseases (18.3%), Ischaemic Heart Disease (12.7%), Lower Respiratory Infections (11.5%) and Diabetes Mellitus (7.2%).

**Conclusions:**

Investigation of registered causes of death using verbal autopsy and medical record review yielded adequate information to enable estimation of cause-specific mortality indicators in Malaysia. Strengthening the national mortality statistics system must be made a priority as it is a core data source for policy and evaluation of the public health and healthcare sectors in Malaysia.

## Background

Reliable vital statistics on births and deaths (including causes of deaths) are important parameters for monitoring health and are routinely used as evidence for health planning [1]. Numbers of death and mortality rates by age, sex and cause are essential indicators for population health assessment. However, in 2005, only about a third of the world’s countries had almost 100% completeness of death registration [2]. Furthermore, only 23 countries were classified to have high-quality registration data on causes of death [2]. Malaysia is one of few Asian countries with long standing and functional vital registration systems. However, the quality of data has been inadequate for routine estimation of key mortality indicators. This is principally due to a persistently high proportion of deaths attributed to non-specific causes of death. Even in 2013, approximately 30% of deaths in the registration data were noted to be from such non-specific causes, which limits the utility of the overall dataset for population health assessment [3].

Currently, there are two methods in place for death certification and registration in Malaysia, based on the place of occurrence of the death. Firstly, deaths in hospitals are certified as to cause by attending physicians, and these are termed as medically-certified deaths. These include deaths from injuries for which forensic post mortem investigations are undertaken to determine the causes of death from medico-legal perspectives. Secondly, deaths that occur at home are reported to the local police station by relatives of the deceased, who also provide a ‘lay’ opinion of the cause, and these deaths are termed as ‘non-medically certified’ deaths. All information from both certification methods are reported to the National Registration Department (*Jabatan Pendaftaran Negara*), where annual mortality datasets are compiled and subsequently transferred to the Department of Statistics, Malaysia (*Jabatan Perangkaan, Malaysia*) for quality control, coding, and analysis. In the past decade, the proportion of deaths that are medically certified has improved from 39.0% in 2000 to 51.5% in 2014 [3].

However, there are several specific problems with data quality, particularly in regard to the registered causes of death from either source. Analysis of the data for 2013 indicated that 12.5% of medically certified deaths were assigned to conditions listed in the chapter titled ‘Symptoms, signs and ill-defined conditions’ in the International Classification of Diseases and Health Related Problems, Tenth Revision (ICD-10) [4]. In addition, 6% of deaths are coded ‘sepsis’, and other smaller percentages of deaths are coded to non-specific conditions such as ‘cardiac arrest’ and ‘respiratory failure’. For the non-medically certified deaths, about 60% are coded to ‘old age’ [5]. On combining the data from the two certification methods, it was found that nearly 30% of all deaths had been assigned such ill-defined or non-specific causes of death. These findings clearly indicate the limited utility of available data on causes of death from registration for health planning, or epidemiological research.

In view of these limitations in the Malaysian Vital Registration (VR) data, this study was designed to apply Medical Record review (MR) and Verbal Autopsy (VA) methods in a national sample of deaths in 2013. The study was commissioned by the Malaysian Ministry of Health in 2014, to quantify the biases in the VR data and utilise the findings to develop national estimates of deaths by age, sex and cause for Malaysia. At that time, the most recent year for which VR data was available was 2013 and was hence chosen as the reference period for the study.

## Methods

### Study design

A cross-sectional study was designed to verify registered causes of death in a nationally representative, multistage stratified cluster sample of deaths that occurred in Malaysia during 2013. The study sample frame comprised the dataset of 142,202 deaths in 2013 by age, sex and cause, available from the Malaysian National Department of Statistics [3].

### Sample size

The sample size was determined according to the approach presented in the article by Begg et al. [6] to estimate the optimal sample size of deaths required for measurement of specific population-based mortality indicators within defined margins of error. The sample size estimation is based on a statistical model which uses inputs of age-specific death rates and income per capita for the study population, to predict a proportionate distribution of cause-patterns of mortality according to three broad cause groups (communicable diseases, non-communicable diseases, and injuries) by age and sex [7]. Begg et al. estimated sample sizes for three population examples, representing three different strata from the World Health Organization’s (WHO) classification of countries according to national mortality characteristics [8]. According to this stratification, Malaysia belongs to the stratum of countries with ‘low child and low adult’ mortality levels [8]. For this stratum, they estimated that a representative sample of approximately 11,000 deaths would enable the measurement of cause-specific mortality rates according to the three broad cause groups by specific 5-year age-sex groups of interest, within a 15% relative standard error [see Table 4 in [6]]. We chose this estimate of sample size for this study.

As a cluster sample is more efficient from logistical aspects, the primary sampling unit was set as the health district of Malaysia, for which a design effect of 1.25 was applied to inflate this sample size to 13,750 deaths. Finally, the research team expected about 10% dropout from the household enquiry into causes of death due to migration, non-availability of respondents, or refusal for participation. As a result, after factoring in this expected loss to follow up; a total sample size of about 15,000 deaths was estimated for this study.

A multistage stratified cluster sampling strategy was used to select 19 out of the total 144 health districts in Malaysia for this study. All deaths that were registered in the selected districts in 2013 were included in the study, and these amounted to a total of 14,497 deaths. The selected sample of deaths was tested and found adequate for national representativeness in terms of age-sex distribution, proportions of medically certified and non-medically certified deaths, and distributions of registered causes of medically and non-medically certified deaths.

### Data collection and processing

For each of the selected districts, the National Registration Department provided a list of all deaths that occurred in the year 2013. For each death, the details of identity, address, reporting institution, and cause of death as determined at registration were given to the District Health Office (DHO) for reinvestigation of the cause of death using two potential approaches. Firstly; all deaths were followed up with a household visit to conduct verbal autopsy (VA) interviews to collect information to ascertain and verify the registered cause(s) of death. Secondly, deaths that had occurred in hospitals were also followed back to review medical records (MR) to verify the registered cause(s) of death. Ethical approval for this study was obtained from the Malaysian Medical Research Ethic Committee with the registration number NMRR-13-1369-18,689. Informed written consent was taken from interviewee before face-to-face interview. Consent was also obtained from the interviewee to review the medical records of the deceased for deaths that had occurred in a medical facility.

For the VA component, details of the development of VA questionnaires, interviewer training and field procedures are provided elsewhere [9]. In brief, international standard VA questionnaires prepared by the WHO [10] and the Population Health Metrics Research Consortium [11] were reviewed and used as a basis to develop a set of questionnaires adapted to the Malaysian context. Following a set program of training, selected verbal autopsy interviewers (attached to the local DHO) were provided with details of the address of the deceased within their health clinic areas. They then visited the home and after obtaining informed consent, conducted a face-to-face interview with a family member of the deceased. VA interviewers were blinded from the cause of death in the registration database, so that the VA interview remained free from bias from this aspect. Completed questionnaires were returned to the DHO, where supervisors reviewed the completed questionnaires for any missing variables or incomplete data. Supervisors also provided field support to staff, where necessary.

All completed questionnaires were submitted to teams of physicians comprising public health specialists and family medicine specialists, for review and assignment of causes of death. Physician reviewers of VA underwent a 3-day training program which covered the principles and practice for assigning causes of death from VA. The training included sessions on diagnostic guidelines for common causes of death, principles of death certification and rules for underlying causes of death, as well as practical exercises and tests of inter-rater reliability to establish consistency between reviewers. Subsequently, each VA questionnaire was reviewed by one physician, who reviewed the questionnaire and assigned causes of death in a format based on the international medical death certificate. In addition, all completed VA questionnaires and death certificates were reviewed by a central team of experienced public health physicians at the Institute for Public Health, Ministry of Health Malaysia, who verified the diagnoses on each VA death certificate, assigned codes from the Tenth revision of the International Classification of Disease and Health Related Problems (ICD-10), and selected the underlying cause of death from VA [4, 12].

As mentioned above, for hospital deaths in the study sample, the second approach involved review of medical records (MR). These deaths were independently followed up for medical record abstraction and review. Lists of deaths from each health institution were prepared, and trained staff reviewed and abstracted information from medical records using a specially designed MR abstraction form. The form included sections to record essential relevant details from the clinical history, findings from physical examination and laboratory and imaging investigations, and details of the clinical course of events culminating in death. Completed MR abstraction forms were reviewed by a member from a panel of medical specialists trained in medical death certification, who certified the cause(s) of death using the standard international medical death certificate. Each form was reviewed by one physician only to determine the cause(s) of death, with the facility to seek a second opinion, if necessary, All completed cause of death certificates (from MR) were reviewed by trained coders, who first assigned ICD-10 codes for each recorded cause of death, and subsequently applied the ICD mortality rules to select the underlying cause for each MR death certificate [4, 12].

Thus, each death in the study sample was assigned a cause of death in the vital registration (VR) data, an underlying cause from the VA investigation, and for those deaths that had occurred in health facilities, an underlying cause from the medical record (MR). The underlying causes coded according to the 3-character ICD code from VR, VA, and MR were then aggregated to the WHO Global Burden of Disease categories [13] for descriptive and comparative analyses.

### Data analysis

The data analysis was done for both arms of the study. For non-hospital deaths, the VA diagnoses were compared with the causes recorded at registration (VR diagnoses), to understand the degree of the reliability of the registration data. Patterns of misclassification between VA and VR diagnoses were assessed, along with the net misclassifications for specific cause categories. These misclassification patterns were analyzed in terms of overall changes in cause-specific mortality proportions for each cause, as determined from the VA study. Eventually, the VA cause-specific mortality fractions for the sample of non-medically certified deaths were adopted for estimating the overall cause-specific patterns for non-medically certified deaths in Malaysia.

Similar analyses were also conducted for the sample of medically certified deaths, for which the diagnoses of underlying causes of death from the MR review were compared with the registration diagnoses. In this analysis, the validity of the registration diagnoses for each cause was assessed in terms of sensitivity, specificity and positive predictive value, using the medical record review diagnoses as the reference standard (data not shown). The net misclassifications for specific cause categories were analysed in terms of overall changes in cause-specific mortality proportions that resulted from the MR review. The MR derived cause-specific mortality fractions were adopted for estimating the overall cause-specific patterns for medically certified deaths in Malaysia.

#### Cause specific mortality estimates

The revised cause-specific mortality proportions for each cause in the hospital death and home death components of the study sample were first applied to the total number of registered deaths in each component separately for males and females, to generate the total estimated number of deaths from each cause. Subsequently, these total numbers of deaths from each cause for each sex were weighted according to the age distributions for each cause as observed in the registration data, separately for hospital and home deaths. This step resulted in the corrected numbers of deaths by age, sex and cause for the hospital and home deaths. These revised death numbers were then summed across the two components to derive the preliminary national mortality estimates by age, sex and cause. Subsequently, standard redistribution algorithms used in the WHO Burden of Disease methodology were applied to redistribute the remaining deaths with ill-defined causes, as well as those with codes from non-specific cardiovascular diseases and cancers without mention of primary site [14].

After corrections for ill-defined and non-specific codes, the resultant estimates were first converted to proportionate distributions by cause for each sex-age category (i.e. the proportionate distribution of causes for deaths in males aged 5–9 years, males age 10–14 years and so on). Subsequently, these proportionate distributions by cause for each sex-age category were fitted to the total national registered numbers of deaths for the corresponding sex-age category, to generate the resultant final estimates of deaths in 2013 by age, sex and cause. These final estimates were analyzed to derive the rank order and magnitude of leading causes of death (for all ages together) by sex at the national level. These rank order estimates were compared with the rank orders from the vital registration data, to understand the influence of the field research on the overall rank structure and proportional magnitude of cause-specific mortality in males and females separately.

#### Directly standardized mortality rates

We also used our study results to estimate direct age standardized death rates (ASDR) for specific causes in males and females. We used national population estimates by age and sex for Malaysia for 2013, obtained from the National Statistics Office [3], to first derive the observed death rates. These were based on the actual deaths we recorded in our sample, and the number of individuals in the local denominator population for each age group scaled down to our sample size. We then used the WHO 2000 population standard [15] as the reference to derive direct ASDRs for each cause by sex. Finally, we used the methodology published by the Association of Public Health Observatories, Public Health England [16] to compute the 95% confidence intervals for the ASDRs The empirically derived Malaysian cause-specific ASDRs by sex for 2013 from our study were compared to imputed estimates of ASDRs derived for Malaysia in 2013 by the Global Burden of Disease 2016 study, conducted by the Institute of Health Metrics and Evaluation, USA [17].

## Results

The total sample for this study was 14,497 deaths, comprising 7487 (51.6%) hospital deaths and 7010 (48.4%) home deaths. Field data collection successfully conducted medical record review for 5041 hospital deaths. VA procedures were successfully completed for 6843 deaths, which included 3724 home deaths and 3119 hospital deaths. The study response rate was 67.0% (ie: at least one of the verification procedures was successfully conducted) but this ranged from 40 to 84% across all the districts.

**Fig. 1:**
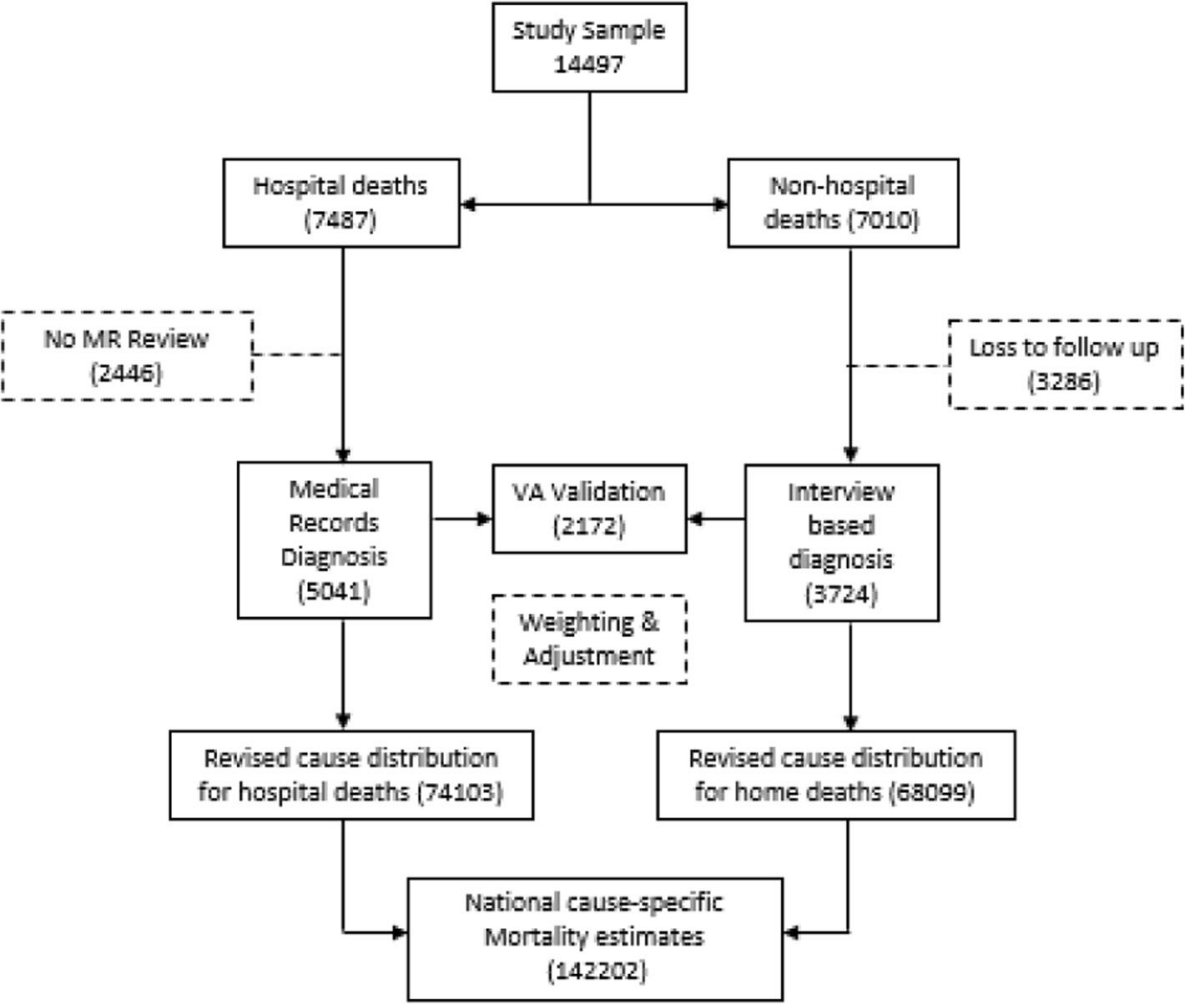
Flow chart of results of data collection and approach to data analysis

On matching the cases across the two study arms, it was found that there were 2172 cases for which both MR review and VA procedures were completed. Overall, the losses to follow up were substantially higher for non-medically certified deaths (47%) as compared to medically-certified deaths (32%). We investigated whether the losses to follow-up as shown in the Fig. 1 resulted in any major bias in the final study sample, from two aspects. Firstly, we compared the age-sex proportionate distributions of the final study sample for hospital and home deaths with the proportionate distributions for each sub group from the vital registration data (see Table 1). As can be seen, there was close approximation between the proportionate distributions, with < 3% difference in proportions across all sub groups.

**Table 1:** Comparison of proportionate distributions of deaths by age and sex between the study sample and vital registration data, Malaysia, 2013

CATEGORY	Age group	MALES		FEMALES		TOTAL	
		Study sample	Vital registration	Study sample	Vital registration	Study sample	Vital registration
Hospital deaths	0–14	5.0%	5.5%	5.4%	6.6%	5.2%	5.9%
	15–59	44.0%	46.5%	35.9%	33.8%	40.9%	41.7%
	60+	50.9%	48.0%	58.7%	59.5%	54.0%	52.3%
TOTAL DEATHS		3078	46,205	1963	27,898	5041	74,103
Home deaths	0–14	1.1%	1.4%	1.6%	1.2%	1.3%	1.3%
	15–59	24.3%	26.0%	14.3%	15.6%	19.6%	21.1%
	60+	74.4%	72.5%	84.1%	83.2%	79.1%	77.6%
TOTAL DEATHS		1948	35,736	1776	32,363	3724	68,099

**Fig. 2:**
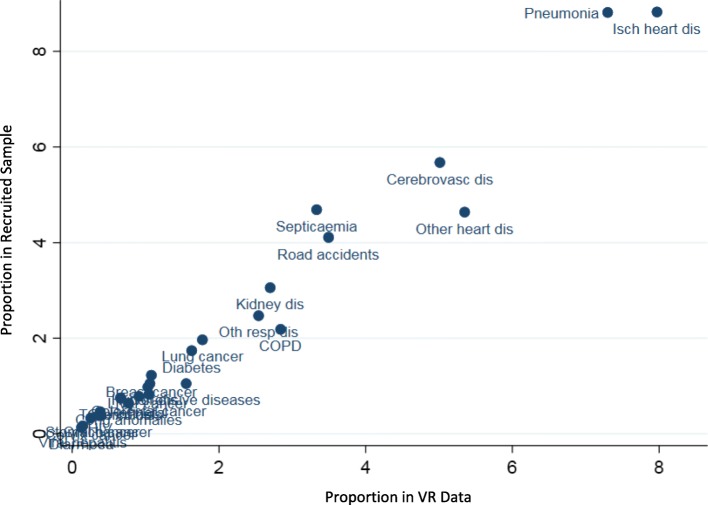
Comparison of proportionate cause distributions of VR and study recruited sample deaths, Malaysia 2013

We also analysed the potential impact of losses to follow up on the distribution of causes of death in the final study sample. Figure 2 below shows a scatter plot with each dot representing the proportion of a specific cause in the vital registration data on the *x-axis*, and its corresponding proportion as per registration diagnosis in the final recruited sample for the field study on the *y-axis*. It can be seen that for the majority of causes, the proportions for VR diagnosis in the national data and in the final recruited sample are very similar (along the 45° line). For ill-defined conditions, the proportion in the VR data was 29.7%, as compared to 32.8% in the final study sample Overall, these findings suggest that the losses to follow up in this study are non-differential according to age, sex and cause distributions.

As described in the methods section, the study findings on reclassification of causes of death from the medically certified and non-medically certified deaths were used to develop adjusted cause-specific mortality estimates for Malaysia. Tables 2 and 3 show the estimated leading causes of death for males and females for 2013 from the study. Ischaemic heart disease emerged as the leading cause of death among males in Malaysia, estimated to cause 12,656 (15.4%) of all male deaths. Cerebrovascular disease and chronic obstructive pulmonary disease are second and third on the list respectively, causing 13.7 and 8.5% of all male deaths. For females, cerebrovascular disease was the leading condition causing 11,057 (18.3%) deaths, followed by Ischaemic heart disease and lower respiratory infections causing 12.7 and 11.5% of deaths respectively.

**Table 2:**
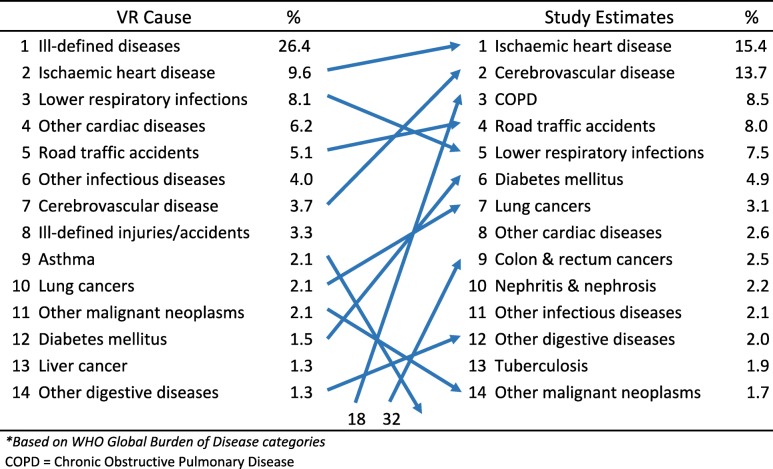
Comparison between leading causes of death^a^ from vital registration data and from the field study estimates for males, Malaysia 2013

^a^ *Based on WHO Global Burden of Disease categories*

*COPD* Chronic Obstructive Pulmonary Disease

**Table 3:**
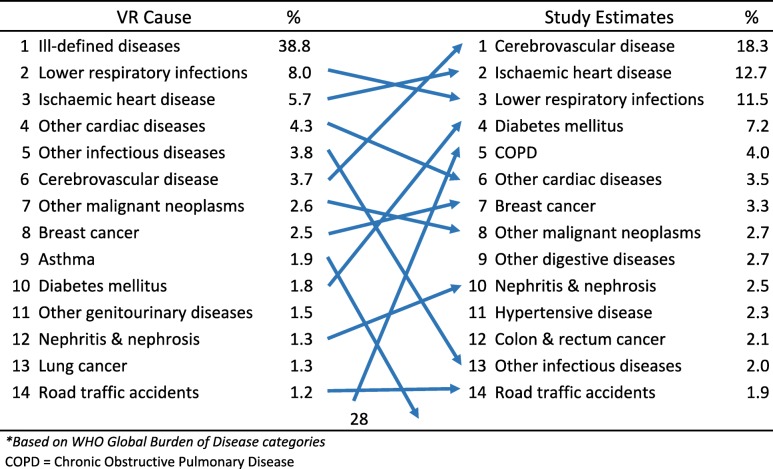
Comparison between leading causes of death^a^ from vital registration data and from the field study estimates for females, Malaysia 2013

^a^ *Based on WHO Global Burden of Disease categories*

*COPD* Chronic Obstructive Pulmonary Disease

The changes between the original registration data and the final study estimates are also shown in Tables 2, 3 and 4. These demonstrate the impact and importance of the field research study. Ill-defined causes are no longer the leading cause of death for both males and females. Overall, marked changes in both rank order and magnitude of proportionate mortality that have resulted from the field research are indicative of the problems with the quality of data on causes of death from the vital registration system. In particular the three-fold increase in proportionate mortality from chronic obstructive pulmonary disease in males, and the four-fold increase from diabetes in both males and females have now highlighted the importance of these causes, and the urgent need for interventions to reduce the premature mortality burden from them. Marked increases in rank order were also observed for lung and colorectal cancers in males.

**Table 4:**
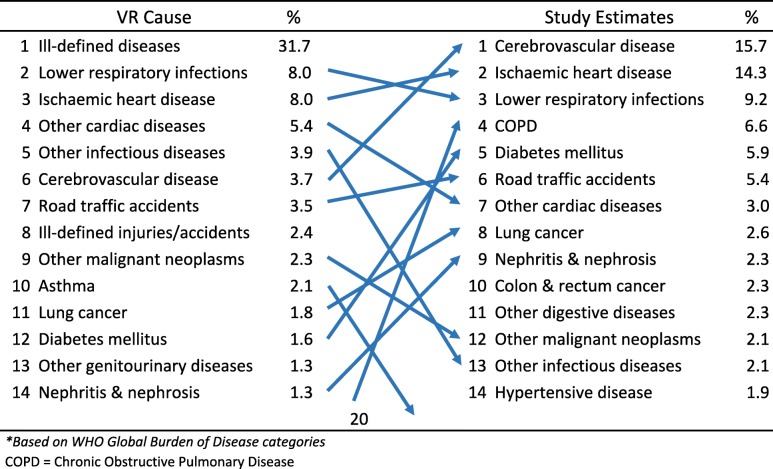
Comparison between leading causes of death^a^ from vital registration data and from the field study estimates for both sexes, Malaysia 2013

^a^ *Based on WHO Global Burden of Disease categories*

*COPD* Chronic Obstructive Pulmonary Disease

From another perspective, comparison of the mortality estimates from this study with national mortality estimates developed for Malaysia in 2013 as part of the Institute of Health Metrics and Evaluation (IHME) Global Burden of Disease (GBD) study [17] also show certain important differences. Tables 5 and 6 display comparisons of age-standardised mortality rates per 100,000 population in 2013 between the study estimates and the IHME GBD estimates for males and females. Taking the study estimates as the reference, the IHME GBD estimated ASDRs are substantially higher or lower than the study estimated ASDRs for several of the leading causes, notably ischaemic heart disease, stroke, chronic obstructive pulmonary disease, and diabetes in both males and females. Similarly, there were significant differences in the IHME GBD estimated ASDRs for tuberculosis and colorectal cancers in males, and for lower respiratory infections and hypertensive disease in females. These comparisons highlight the potential differences arising from the analytical approach of using local empirical data for mortality estimation as in this study, as compared to the statistical modelling approach to mortality estimation, as used in the IHME GBD study.

**Table 5:** Age-standardised death rates per 100000 population for leading causes of death as estimated from this study and the IHME Global Burden of Disease study for males in Malaysia, 2013

Cause of death	Study		GBD 2013		% difference
	ASDR	95% CI	ASDR	95% CI	
Ischaemic heart disease	102.7	95.5, 110.2	140.3	129.8, 151.6	36.6%
Cerebrovascular disease	92.1	85.2, 99.4	50.0	44.9, 55.3	−45.7%
Chronic obstructive pulmonary disease	60.6	54.9, 66.7	23.1	20.3, 26.8	−61.8%
Lower respiratory infections	49.0	44.0, 54.4	52.5	30.7, 63.8	7.1%
Road traffic accidents	42.8	38.6, 47.4	34.5	30.3, 38.4	−19.5%
Diabetes mellitus	34.0	29.9, 38.5	13.9	12.3, 15.7	−59.2%
Lung cancer	22.6	19.2, 26.4	21.3	18.8, 24.4	−5.7%
Colon and rectum cancers	17.6	14.6, 20.9	10.1	8.9, 11.5	−42.6%
Chronic kidney disease	14.1	11.4, 17.0	12.1	10.6, 13.7	−14.5%
Tuberculosis	12.0	9.6, 14.7	7.1	6.1, 8.2	−40.9%

+ ve % difference indicates higher estimate by GBD, −ve % difference indicates lower estimate by GBD

**Table 6:** Age-standardised death rates per 100,000 population for leading causes of death as estimated from this study and the IHME Global Burden of Disease study for females in Malaysia, 2013

Cause of death	Study		GBD 2013		% difference
	ASDR	95% CI	ASDR	95% CI	
Cerebrovascular disease	105.2	97.3, 113.5	41.7	37.1, 46.5	−60.3%
Ischaemic heart disease	71.4	65.0, 78.3	86.3	78.3, 96.9	20.8%
Lower respiratory infections	67.2	60.8, 74.1	39.5	21.2, 49.5	−41.3%
Diabetes mellitus	38.4	33.8, 43.2	14.9	12.9, 17.2	−61.2%
Chronic obstructive pulmonary disease	24.0	20.1, 28.1	8.5	7.1, 10.8	−64.5%
Breast cancer	16.0	13.2, 19.0	13.2	11.3, 15.2	−17.5%
Chronic kidney disease	14.0	11.2, 17.2	10.9	9.6, 12.2	−22.4%
Hypertensive disease	13.0	10.2, 16.0	1.8	1.4, 2.6	−86.1%
Colon and rectum cancers	11.3	8.9, 14.1	7.4	6.4, 8.5	−34.2%
Lung cancer	10.4	8.1, 13.1	8.8	7.6, 9.9	−15.5%

+ ve % difference indicates higher estimate by GBD, −ve % difference indicates lower estimate by GBD

## Discussion

In view of the limitations in the quality of data on causes of death from the vital registration system in Malaysia, this study was designed to conduct a thorough verification of registered causes in a national sample of deaths that occurred in 2013. The results from the verification were used to derive corrected estimates of cause-specific mortality in Malaysia. The mortality estimates for 2013 from this study are clearly more useable than registration data, for health policy, monitoring and research. Ill-defined causes are no longer the leading cause of death, and the verification procedures from the field research have resulted in a large-scale empirical reassignment of these deaths to more specific causes. Furthermore, the reassignment has resulted in major changes to the leading causes of death among males and females. Not only are we more accurately able to quantify the burden of the leading causes of death, but also the changes seen in diseases such as diabetes and chronic obstructive lung disease would clearly highlight the importance of prioritizing, managing and preventing these diseases at all levels.

A similar study to verify a sample of registered causes of death and utilize the findings to develop national cause-specific mortality estimates was conducted in Thailand for 2005 [18, 19]. The Thai study identified that verbal autopsy was found useful to understanding cause specific mortality at population level. Over the past decade, VA had been used as a method to determine cause of deaths in places where majority of deaths occur without medical supervision [20]. It has been used for deaths in various age groups [21, 22] and also across a range of causes of death [23–25]. In Brazil, verbal autopsy has been successfully implemented to improve the accuracy of deaths originally classified to ill-defined causes in the vital registration system [26]. The experience from this study indicates that it is feasible to use VA to improve cause of death reporting in Malaysia and would help increase the accuracy of the mortality statistics for home deaths reported to the national vital registration system. A recent qualitative study also demonstrated a high level of community support in Malaysia for verbal autopsies [27]. From another perspective, the study findings also indicate weaknesses in the quality of medical certification of causes for hospital deaths. Several international studies have also indicated the importance of medical record review to improve the quality of cause of hospital deaths in developing countries [28–31]. Hence, death certification and coding for hospital deaths in Malaysia should also be strengthened in order to increase the overall accuracy of national mortality statistics from vital registration.

Despite the positive outcomes in terms of more reliable estimates, as well as the strengths of the analytical approach used in this study, there are certain limitations. Firstly, retrospective data collection is susceptible to losses to follow-up, as observed in this study. In many of the instances of failure to conduct VA interviews, the primary caregiver of the deceased had moved residence to a different location or could not be contacted despite three attempts. Further, there were cases where the deceased had been living alone, with no surviving relative. Consequently, the VA interview could not be conducted. Finally, there were a few instances of incomplete recording of the address of the deceased in the registration data. In the study sample for hospital deaths, MR review could not be completed in three hospitals, owing to non-availability of manpower to complete this task during the study period. Fortunately, additional analysis, as described in Table 1 and Fig. 2, suggest that the losses to follow up in this study were largely non-differential in regard to age, sex and cause distributions, hence minimising any potential biases in the study findings from this aspect.

Other potential sources of bias include data collection errors during verbal autopsy interviews, arising from inaccurate information provided by family members who might not have been the primary caregiver of the deceased during the final illness. In addition, verbal autopsy is also prone to recall bias as a result of poor memory on the part of the respondent. Difficulty in recalling the symptoms and illness prior to death by relatives increases with the length of time since death. Since the VA interviews were done for deaths that had occurred about 12 months before the date of interview, the large gap between time of death and the VA interview potentially introduces recall bias. Finally, bias could also occur on account of a deliberate pattern of mis-reporting of symptoms by the interviewee, on account of administrative pressures or social stigma, notably in the event of injury causes with medico-legal implications, or from diseases such as HIV/AIDS or tuberculosis. As mentioned earlier, while the study implementation, quality control measures, and data analysis have included provisions to ensure accuracy of study findings, these limitations should be borne in mind.

From a policy perspective, the marked differences between the estimated mortality rates from this study and similar estimates for Malaysia in 2013 from the IHME GBD 2013 Study require careful attention. The estimates derived from this study are based on field research, which involved rigorous processes for data collection, management and analysis, as described in this article. These field research methods create confidence in the resultant mortality estimates, and a sense of political relevance and ownership which enhance their utilisation for national health policy and research. In contrast, the IHME GBD estimates for several causes are based on statistical models constructed from a dataset of mortality and cause of death patterns from a wide range of countries across the world [32] and may not always be applicable to the Malaysian context. Also, the estimation methods apply model-based reassignment strategies for deaths originally assigned ill-defined and non-specific codes in national datasets, as opposed to the empirical reassignment strategies adopted for our study estimates [33]. The IHME GBD model-based estimates for Malaysia could be biased from these aspects. Future mortality estimation exercises for Malaysia could take into account the findings from the research reported here as local inputs for the statistical models which have been extensively used in more recent analyses reported by the GBD Study Collaborators [34–38].

Inaccurate mortality statistics from vital registration have an extensive and often unquantifiable negative implications for national health care systems. While this study provides the first ever Malaysian national mortality estimates derived from empirical local data, Malaysia needs to pay close attention to strengthening the performance of its civil registration and vital statistics system. The research experience from this study, along with the critical mass of human and technical resources developed in hospitals, health clinics, district health offices and the Institute of Public Health should be utilised in devising interventions to improve the quality of registered causes of death.

## Conclusion

This study highlights that there is room for and a need to strengthen the mortality statistics system in Malaysia, and this should be made a priority. The verbal autopsy and medical record review methods applied in this study and its findings have improved our understanding of mortality patterns in the country. Verbal autopsy methods should be built into routine practice and scaled up across the country, to improve registration data. Training on medical certification of causes of death should be regularly provided in all hospitals. Studies similar to this one should also be conducted on a periodic basis to improve the empirical basis for cause of death estimation in Malaysia and enable a better understanding of the epidemiological transition that occurs at national and sub national level across the country, and better plan the future health needs of the nation.

## Abbreviations

DHO: District Health Office
ICD: International Classification of Disease and Related Health Problems
MR: Medical Records Review
VA: Verbal Autopsy
VR: Vital Registration
WHO: World Health Organization
